# The Relationship Between the Remnant Cholesterol Inflammation Index and Coronary Collateral Circulation in Patients with Chronic Total Occlusion

**DOI:** 10.3390/jcm15197352

**Published:** 2026-09-22

**Authors:** Erdoğan Yaşar, İbrahim Aktaş, Tolga Çakmak, Furkan Ay

**Affiliations:** 1 Department of Cardiology, Malatya Training and Research Hospital, 44090 Malatya, Türkiye; 2 Department of Cardiology, Faculty of Medicine, Malatya Turgut Özal University, 44090 Malatya, Türkiye; ibrahim.aktas@ozal.edu.tr (İ.A.); furkanay123456@gmail.com (F.A.); 3 Department of Cardiology, Faculty of Medicine, Balıkesir Atatürk State Hospital University of Health Sciences, 10100 Balıkesir, Türkiye; tolgacakmak85@gmail.com

**Keywords:** chronic total occlusion, coronary collateral circulation, remnant cholesterol inflammation index

## Abstract

**Background and Objectives:** Although chronic total occlusion (CTO) often presents with varying degrees of coronary collateral circulation (CCC), the biological factors accompanying inadequate collateralization are not fully established. The Remnant Cholesterol Inflammation Index (RCII) is a composite metric integrating atherogenic lipid load and systemic inflammation. This study examined the association between RCII and CCC and the discriminative ability of RCII for identifying insufficient collateral networks in patients with CTO. **Materials and Methods:** This retrospective, single-center analysis evaluated 359 patients with at least one major epicardial CTO who underwent diagnostic coronary angiography between 15 January 2023 and 15 January 2026. Participants were stratified via the Rentrop scoring system into poor (grades 0–1; *n* = 168) and good (grades 2–3; *n* = 191) CCC cohorts. RCII was calculated as [remnant cholesterol (mg/dL) × high-sensitivity C-reactive protein (hs-CRP, mg/L)]/10, with remnant cholesterol derived as total cholesterol minus LDL-C and HDL-C. Univariable and multivariable logistic regression models, together with receiver operating characteristic (ROC) curve analysis, were used to assess the association of RCII with poor CCC and its discriminative ability. **Results:** Patients with poor CCC exhibited significantly higher RCII values compared with the good CCC cohort (19.7 ± 4.7 vs. 6.5 ± 2.4, *p* < 0.001). Following multivariable adjustment, higher RCII remained independently associated with poor collateralization (OR 1.120, 95% CI 1.052–1.199; *p* < 0.001), alongside right coronary artery (RCA) CTO and the systemic immune-inflammation index (SII). RCII showed discriminative ability for poor CCC (AUC 0.792, 95% CI 0.745–0.835; *p* < 0.001), which was greater than that of remnant cholesterol, hs-CRP, or SII considered separately. In this cohort, an RCII threshold above 12.3 identified poor CCC with 80% sensitivity and 71% specificity. **Conclusions:** In this retrospective cohort, higher RCII was independently associated with inadequate coronary collateralization in the setting of CTO. RCII may represent a candidate marker of the combined residual lipid and inflammatory burden; prospective studies and external validation, together with formal assessment of clinical utility, are required before any clinical application can be considered.

## 1. Introduction

Coronary artery disease (CAD) remains a leading cause of cardiovascular morbidity and mortality worldwide [1]. Chronic total occlusion (CTO), defined as a complete coronary artery obstruction with TIMI grade 0 flow persisting for at least three months, represents an advanced stage of CAD, observed in approximately 15% to 30% of patients undergoing coronary angiography [2]. In this setting, the coronary collateral circulation (CCC) acts as a vital adaptive mechanism by providing an alternative blood supply to the ischemic myocardium [3]. The extent of collateralization varies significantly among individuals. Well-developed collaterals (Rentrop grades 2–3) are associated with better clinical outcomes by limiting the extent of myocardial infarction, preserving left ventricular function and reducing symptoms [4,5]. In contrast, inadequate collateral development is associated with more extensive myocardial damage and a poor prognosis. However, collateral development shows marked heterogeneity between individuals, and the mechanisms underlying this variation have not yet been fully elucidated [6].

The extent of coronary collateralization is determined by the interplay of vascular growth, endothelial responses, inflammation, and haemodynamic forces. Arteriogenesis and angiogenesis are regulated by multiple mediators, including vascular endothelial growth factor (VEGF), hypoxia-inducible factor-1α (HIF-1α), and endothelial progenitor cells, which contribute to vascular adaptation under chronic ischemic conditions [7]. Accumulating evidence indicates that systemic inflammation, endothelial dysfunction, and oxidative stress are associated with less effective collateralization [7,8]. Consequently, several systemic inflammatory indices—including the systemic immune-inflammation index (SII), neutrophil-to-lymphocyte ratio (NLR), and neutrophil percentage-to-albumin ratio (NPAR)—have been reported to be independently associated with inadequate collateral development in CTO patients [9,10,11,12]. Collectively, these findings suggest that inflammation is one of the pathophysiological factors accompanying limited collateral circulation.

Remnant cholesterol (RC), representing the cholesterol content within triglyceride-rich lipoproteins, is increasingly recognized as a major contributor to residual atherosclerotic cardiovascular risk [13,14]. Unlike low-density lipoprotein (LDL) cholesterol, remnant particles can penetrate the arterial intima without requiring prior oxidation and are directly engulfed by macrophages. This triggers rapid foam cell formation, exacerbating local inflammatory responses and endothelial dysfunction [15]. Notably, elevated remnant-like particle cholesterol concentrations have previously been linked to impaired collateral maturation in patients with CTO [16]. Given that lipid accumulation and inflammation are closely linked in atherogenesis, evaluating either pathway in isolation may yield an incomplete pathophysiological picture of collateral vessel formation.

The Remnant Cholesterol Inflammation Index (RCII) has recently been introduced as a composite biomarker integrating both atherogenic lipid burden and systemic inflammatory activity. By combining these parameters, RCII may capture the joint contribution of residual lipid risk and inflammatory status more comprehensively than isolated markers. Recent studies have reported that RCII is independently associated with myocardial infarction, stroke, and all-cause mortality [17,18,19]. Furthermore, RCII has shown greater discriminative ability than RC alone or standard inflammatory markers in relation to microvascular dysfunction [20]. These data suggest that RCII may serve as an accessible indicator of the combined lipid and inflammatory burden.

Despite these observations, the relationship between RCII and macrovascular coronary collateralization in patients with CTO has not, to our knowledge, been specifically investigated. Therefore, we aimed to investigate the association between RCII and CCC and to evaluate the discriminative ability of RCII for identifying patients with poor collateral development (Rentrop grades 0–1).

## 2. Materials and Methods

### 2.1. Study Design and Study Population

This retrospective, single-center observational cohort study was conducted at the Cardiology Clinic of Malatya Training and Research Hospital. We screened adults aged ≥18 years who underwent coronary angiographic evaluation at our institution between 15 January 2023, and 15 November 2025. The medical records of eligible patients during this period were retrospectively reviewed. Data organization, data cleaning, and statistical analysis were performed during the subsequent two-month period, from 15 November 2025, to 15 January 2026. Patients were considered eligible when coronary angiography demonstrated a chronic total occlusion (CTO) involving at least one major epicardial vessel. Out of 592 initially evaluated patients, 233 were excluded for failing to meet the inclusion criteria. The final cohort was stratified into two groups based on the Rentrop collateral grading system: the poor coronary collateral circulation (CCC) group, comprising Rentrop grades 0–1 (*n* = 168), and the good CCC group, comprising Rentrop grades 2–3 (*n* = 191).

The study protocol received approval from the Clinical Research Ethics Committee of Malatya Turgut Özal University (Approval No. 2025/452). The study was performed in accordance with the Declaration of Helsinki. Because the study was retrospective in nature, the requirement for written informed consent was waived by the ethics committee.

Exclusion criteria were defined as follows: (1) acute coronary syndrome episode within the preceding three months; (2) active infectious disease, known autoimmune conditions, or chronic systemic inflammatory disorders; (3) severe renal dysfunction, defined as an eGFR below 30 mL/min/1.73 m^2^; (4) active malignancy; (5) moderate-to-severe valvular heart pathology or a history of coronary artery bypass grafting; (6) systolic heart failure characterized by a left ventricular ejection fraction (LVEF) < 40%; (7) hematological dyscrasias or ongoing therapy with systemic corticosteroids or immunosuppressants; (8) prescription of lipid-lowering agents within the three months prior to admission; and (9) missing or incomplete baseline clinical and laboratory documentation. The study flow is shown in Figure 1.

### 2.2. Coronary Angiography and Assessment of Coronary Collateral Circulation

Diagnostic coronary angiography was systematically performed utilizing the standard Judkins technique via either radial or femoral access, dictated by operator discretion. To ensure objective evaluation, angiographic sequences were independently reviewed by two experienced cardiologists who were blinded to the baseline clinical and biochemical profiles of the patients. In instances of interobserver discrepancy regarding collateral grading, the images were reevaluated by a third senior interventional cardiologist to reach a final consensus. A CTO was defined as an absolute occlusion of a coronary artery resulting in Thrombolysis in Myocardial Infarction (TIMI) grade 0 flow. This occlusion had to be angiographically evident in a minimum of two orthogonal projections. The chronicity of the occlusion (estimated at ≥3 months) was validated through historical angiographic data, stable clinical symptom duration, or specific morphological vessel characteristics indicating chronic obstruction. Collateral network robustness was quantified via the Rentrop classification system:

Grade 0: Absence of angiographically visible collateral flow.

Grade 1: Filling of the side branches originating from the occluded vessel, lacking opacification of the epicardial segment.

Grade 2: Partial angiographic opacification of the epicardial segment via collateral conduits.

Grade 3: Complete opacification of the distal epicardial artery sustained entirely by collateral channels.

For statistical dichotomization, Rentrop grades 0–1 constituted the poor CCC cohort, whereas grades 2–3 formed the good CCC cohort. In multi-vessel CTO presentations, the occlusion subtending the most extensive myocardial territory was targeted for collateral grading.

### 2.3. Clinical Characteristics and Definitions

Baseline demographic parameters (age, sex), established cardiovascular risk factors (hypertension, diabetes mellitus, dyslipidemia, smoking habits), and current pharmacotherapy regimens were systematically extracted from institutional electronic health records. Antiplatelet therapy was not included as an analytical variable because a substantial proportion of patients were newly diagnosed with CTO at the time of coronary angiography and had not yet initiated antiplatelet treatment. Hypertension was diagnosed if the resting systolic/diastolic blood pressure was ≥140/90 mmHg or if the patient was actively managed with antihypertensive medications, aligning with the 2024 ESC Hypertension Guidelines [21]. Diabetes mellitus was documented according to the 2025 American Diabetes Association (ADA) Standards of Care [22], defined by a fasting plasma glucose ≥126 mg/dL, an HbA1c level ≥6.5%, or the prescription of antidiabetic pharmacotherapy. Smoking history was classified based on active tobacco consumption at the time of the procedure.

### 2.4. Laboratory Measurements

Peripheral venous blood sampling was conducted on the morning of the angiographic procedure, following a minimum 12-h overnight fasting period. The specimens were promptly analyzed in the institutional central laboratory employing standardized automated diagnostic platforms. The biochemical panel incorporated fasting plasma glucose, HbA1c, serum creatinine, total cholesterol (TC), triglycerides (TG), high-density lipoprotein cholesterol (HDL-C), low-density lipoprotein cholesterol (LDL-C), serum albumin, and high-sensitivity C-reactive protein (hs-CRP). Concurrently, a complete blood count provided absolute values for white blood cells, neutrophils, lymphocytes, monocytes, and platelets. LDL-C levels were derived via the Friedewald formula for samples with triglyceride concentrations <400 mg/dL. Direct LDL-C measurement assays were used if triglyceride levels were ≥400 mg/dL or when directly measured baseline values were available.

Remnant cholesterol (RC) was calculated from the standard lipid profile as the difference between total cholesterol and the sum of LDL-C and HDL-C [23]. The Remnant Cholesterol Inflammation Index (RCII) was calculated as [RC (mg/dL) × hs-CRP (mg/L)]/10, as previously described [24]. NLR, SII, and NPAR were calculated according to previously established definitions [9,10,11].

### 2.5. Echocardiographic Assessment

Prior to angiographic intervention, all patients underwent a comprehensive transthoracic echocardiographic examination utilizing commercially available ultrasound systems. The left ventricular ejection fraction was quantified using the modified biplane Simpson’s rule. The standard assessment protocol documented cardiac chamber dimensions, regional wall motion anomalies, and valvular morphologic defects. Individuals exhibiting moderate-to-severe valvular dysfunction or significant systolic impairment (LVEF < 40%) were excluded from the final cohort.

### 2.6. Statistical Analysis

Statistical analyses were conducted using SPSS Statistics version 26.0 (IBM Corp., Armonk, NY, USA) and MedCalc Statistical Software version 23.2 (MedCalc Software Ltd., Ostend, Belgium). The distribution of continuous variables was assessed with the Kolmogorov–Smirnov test. Variables showing an approximately normal distribution are expressed as mean ± standard deviation, whereas non-normally distributed variables are presented as median values with the interquartile range (IQR). Categorical variables are reported as numbers and percentages. Comparative analyses between the cohorts utilized the independent-samples *t*-test or the Mann–Whitney U test for continuous data, contingent upon distribution normality. Categorical variables were cross-tabulated and analyzed using the chi-squared test.

The association between RCII and poor collateralization was initially examined through univariable logistic regression. Variables yielding a *p*-value < 0.05 univariably, together with major clinically relevant covariates, were advanced into multivariable modeling. The strength of association was expressed as odds ratios (ORs) with 95% confidence intervals (CIs). To avoid multicollinearity arising from the simultaneous inclusion of the composite RCII and its constituents, two distinct multivariable logistic regression models were constructed. Model 1 included age, hypertension, diabetes mellitus, RCA CTO, SII, remnant cholesterol, and hs-CRP. Model 2 included age, hypertension, diabetes mellitus, RCA CTO, SII, and RCII, with RCII replacing remnant cholesterol and hs-CRP to avoid mathematical overlap between the composite index and its individual components. Multicollinearity was assessed using variance inflation factors (VIFs), and all VIF values were <5. The discriminative ability of RCII and of the comparator inflammatory biomarkers for poor CCC was assessed using receiver operating characteristic (ROC) curves. Optimal cut-off values were determined using the Youden index, and the respective areas under the curve (AUC) were compared using the DeLong method. All inferential testing was two-tailed, with the threshold for statistical significance set at *p* < 0.05.

## 3. Results

The final analysis comprised 359 patients with CTO. Poor CCC was identified in 168 patients (46.8%), whereas 191 patients (53.2%) had good CCC. Baseline demographic, clinical, and angiographic characteristics of the study population are presented in Table 1.

Baseline characteristics were broadly comparable between the poor and good CCC groups. No significant between-group differences were observed for age, sex, body mass index, hypertension, smoking status, systolic or diastolic blood pressure, left ventricular ejection fraction, or the extent of coronary artery disease (all *p* > 0.05). Diabetes mellitus was numerically more frequent in the poor CCC group than in the good CCC group; however, this difference did not reach statistical significance (35.1% vs. 27.2%, *p* = 0.106).

The distribution of CTO location also differed between the groups. RCA CTO was more frequent in patients with good CCC than in those with poor CCC (64.4% vs. 50.6%, *p* = 0.008), whereas the frequencies of LAD and Cx CTO were comparable between the groups. Laboratory and inflammatory parameters are summarized in Table 2.

Several metabolic and inflammatory parameters differed according to collateral status. Fasting glucose, HbA1c, total cholesterol, LDL cholesterol, triglycerides, neutrophil and platelet counts, remnant cholesterol, hs-CRP, NLR, SII, and RCII were higher in patients with poor CCC than in those with good CCC (all *p* < 0.05). HDL cholesterol showed the opposite pattern, with lower concentrations observed in the poor CCC group. Serum creatinine, hemoglobin, and lymphocyte counts did not differ significantly between the groups. The between-group difference was most pronounced for RCII, with mean values of 19.7 ± 4.7 in patients with poor CCC compared with 6.5 ± 2.4 in those with good CCC (*p* < 0.001). The regression analysis findings are presented in Table 3.

The univariable analyses identified RCA CTO, remnant cholesterol, hs-CRP, SII, and RCII as variables associated with poor CCC, whereas diabetes mellitus was not significantly associated with collateral status (OR 1.447; 95% CI 0.925–2.264; *p* = 0.106) and was retained in the multivariable models only as a clinically relevant covariate. Because RCII incorporates both remnant cholesterol and hs-CRP, these components were not entered into the same multivariable model as RCII. Accordingly, two separate models were evaluated. Model 1 included RC and hs-CRP together with the other clinical covariates. In this model, RC (OR 1.228; 95% CI 1.004–1.506; *p* = 0.046) and hs-CRP (OR 2.018; 95% CI 1.350–3.007; *p* < 0.001) remained significantly associated with poor CCC, together with RCA CTO (OR 0.562; 95% CI 0.335–0.937; *p* = 0.026) and SII (OR 1.004; 95% CI 1.001–1.007; *p* = 0.009), whereas diabetes mellitus was not independently associated with collateral status (OR 1.008; 95% CI 0.730–1.365; *p* = 0.960). Model 2 replaced the individual components with the composite RCII. In this model, higher RCII was independently associated with poor CCC (OR 1.120; 95% CI 1.052–1.199; *p* < 0.001), while RCA CTO (OR 0.695; 95% CI 0.490–0.990; *p* = 0.043) and SII (OR 1.003; 95% CI 1.001–1.005; *p* = 0.003) also remained significant. Diabetes mellitus again showed no independent association with poor CCC (OR 1.112; 95% CI 0.833–1.496; *p* = 0.450). The ability of RCII to distinguish patients with poor CCC was further assessed by ROC curve analysis. The resulting AUC was 0.792 (95% CI 0.745–0.835; *p* < 0.001). Using a threshold of >12.3, RCII identified poor CCC with 80% sensitivity and 71% specificity (Figure 2).

The AUC was 0.710 (95% CI 0.650–0.770) for SII, 0.687 (95% CI 0.610–0.765) for hs-CRP, and 0.668 (95% CI 0.594–0.735) for remnant cholesterol. Pairwise DeLong comparisons indicated that RCII had greater discriminative ability than remnant cholesterol (*p* = 0.030), hs-CRP (*p* = 0.028), or SII (*p* = 0.035).

## 4. Discussion

In this retrospective cohort of patients with chronic total occlusion (CTO), higher remnant cholesterol inflammation index (RCII) values were independently associated with poorer angiographic coronary collateral circulation (CCC), assessed using the Rentrop grading system. Higher RCII values were associated with less well-developed collateral circulation, and this association persisted after adjustment for traditional cardiovascular risk factors. Because RCII and collateral grade were assessed contemporaneously in a retrospective design, these findings describe an association only; they do not indicate that RCII inhibits collateral formation or that it predicts subsequent collateral development. Interpreted within these limits, RCII, a composite parameter reflecting both remnant cholesterol (RC) and inflammatory activity (hs-CRP), may represent an accessible candidate marker of the atheroinflammatory milieu accompanying less well-developed collateral circulation.

Coronary collateral circulation represents one of the most important adaptive responses to chronic myocardial ischemia. Well-developed collateral vessels preserve myocardial viability, limit infarct size, improve left ventricular function, and are associated with better long-term clinical outcomes [3,4]. However, this process exhibits marked heterogeneity between individuals, and the underlying mechanisms have not yet been fully elucidated [6,25]. Collateral development, or arteriogenesis, is a complex process that develops in response to the pressure gradient and endothelial shear stress arising following occlusion; the progression of this process is closely related to vascular endothelial growth factor (VEGF) signalling, nitric oxide (NO) bioavailability, and the integrity of endothelial function [7,26]. Systemic inflammatory activation may interfere with this process at several levels. In clinical CTO cohorts, composite inflammatory indices are consistently associated with poorer angiographic collateral grades [9,10,11,12,27]. At the cellular level, proteolytic enzymes and pro-inflammatory mediators released by neutrophils contribute to endothelial injury, while lymphopenia associated with chronic inflammation has been linked to reduced VEGF availability and impaired maturation of collateral vessels [28].

hs-CRP, one of the components of RCII, is not merely a marker of inflammation but may also contribute actively to endothelial dysfunction by reducing eNOS activity and increasing oxidative stress [29]. Against this pathophysiological backdrop, the relationship between collateral development and various inflammatory indices in patients with CTO has been investigated intensively in recent years. Parameters such as SII, NLR, the pan-immune-inflammation value (PIV), and NPAR have been reported to be independently associated with poor collateral development [9,10,11,12,27]. In particular, Zhang et al. reported that PIV was independently associated with poor collateral development [27], and Toprak and Karataş recently compared PIV with conventional inflammation-based markers in a CTO population and observed a comparable pattern [12]. However, these indices reflect cellular inflammation parameters alone and do not account for the atherogenic lipid burden. RCII differs in this respect, integrating atherogenic lipid metabolism and systemic inflammation into a single formula.

The value of composite indices derived from routinely available laboratory parameters is not confined to the coronary collateral circulation. In patients with acute coronary syndrome, Eyiol et al. reported associations between several composite inflammatory and nutritional indices and in-hospital mortality, with SII showing the strongest discriminatory performance [30]. In a subsequent multicentre study, PIV and HALP were also independently associated with in-hospital mortality, although their incremental contribution to the TIMI risk score was modest [31]. These observations are directly relevant to the interpretation of our own results, as they support the concept that composite indices derived from routinely available laboratory parameters can capture clinically relevant biological information. In our study, RCII similarly demonstrated greater discriminatory performance than SII, hs-CRP, and remnant cholesterol, suggesting that combining lipid and inflammatory information may provide a useful marker of the biological milieu associated with coronary collateral development.

The pathophysiological role of RC, the lipid component of RCII, is also relevant to processes that adversely affect collateral vessel development. Unlike LDL cholesterol, RC is a highly atherogenic lipoprotein fraction which can penetrate the arterial wall without requiring oxidation and, upon direct phagocytosis by macrophages, triggers an inflammatory cascade [14,32]. RC particles accumulating in the arterial wall intensify local inflammation by increasing the release of mediators such as monocyte chemotactic protein-1 (MCP-1) and may disrupt angiogenesis [14,16]. This process also triggers foam cell formation, fuelling low-grade systemic inflammation and creating an inflammatory burden that parallels CRP levels [32]. Another mechanism of potential relevance to collateral vessel development is the effect of RC on endothelial progenitor cells (EPCs). By accelerating EPC senescence via increased reactive oxygen species, RC has been shown to reduce the capacity of these cells for adhesion, migration, and proliferation [32], which may weaken the cellular substrate of adaptive collateral growth. Consistent with this, Nakamura et al. demonstrated an adverse relationship between RC levels and endothelial vasomotor function, whilst Gao et al. reported that estimated remnant-like particle cholesterol (RLP-C) levels were independently associated with poor collateralisation in patients with CTO, with a stepwise gradient across increasing RLP-C levels [16,33]. Our observation that RCII—which includes RC as a key component—is inversely associated with collateral grade is in keeping with these reports, although the cross-sectional design does not allow the relative contributions of lipid burden and inflammation to be separated.

RCII combines two biologically distinct but closely interconnected components: residual atherogenic lipid burden represented by RC and low-grade systemic inflammation represented by hs-CRP. The clinical relevance of this combination is supported by previous population-based studies showing that concurrent elevations in RC and CRP are associated with an increased risk of cardiovascular events and mortality [17,18]. In the Copenhagen General Population Study, which included more than 103,000 individuals, Doi et al. reported a substantially higher cardiovascular risk among individuals with elevations in both RC and CRP, with a hazard ratio of approximately 1.9 compared with individuals without simultaneous elevation of these markers [17]. In separate population cohorts, Wang et al. and Chen et al. reported that RCII was independently associated with all-cause mortality and with stroke risk, respectively [19,24]. In a separate cohort of patients with Cardiac Syndrome X, we previously observed an independent association between RCII and microvascular dysfunction, with discriminative value beyond that of individual lipid or inflammatory markers, suggesting that the biological relevance of this composite index may extend across different manifestations of coronary vascular disease [20]. Taken together, these findings provide a plausible framework for interpreting the present results: the simultaneous presence of residual atherogenic lipid burden and systemic inflammation may characterise a vascular phenotype in which collateral adaptation is less well developed.

Additionally, anatomical and haemodynamic factors play a role in collateralisation. In our cohort, right coronary artery (RCA) CTO was observed more frequently in patients with good collateral circulation. This finding is consistent with previous reports suggesting that RCA occlusions tend to develop more robust collateral vessels because of favourable anatomical and haemodynamic characteristics. Nevertheless, RCA CTO remained in the multivariable model, and RCII remained independently associated with poor collateral circulation after adjustment for CTO location, indicating that the observed association was not explained by lesion location alone.

From a practical standpoint, RCII is a non-invasive index that can be calculated at no additional cost from laboratory parameters obtained in routine practice. In the present cross-sectional setting, it separated patients with poor and good angiographic collateral grades with moderate discriminative ability. Whether RCII adds information beyond established clinical and angiographic assessment, and whether it has any bearing on treatment decisions, cannot be determined from these data. Such questions would require prospective studies with clinical endpoints and formal clinical-utility analyses, and no recommendation regarding medical therapy or revascularisation strategy can be derived from the present findings.

Several limitations of the present study should be acknowledged. First, its single-center, retrospective observational design precludes causal inferences. Second, RCII and collateral grade were assessed contemporaneously from a single baseline measurement; the analysis is therefore cross-sectional and cannot establish whether RCII precedes, follows, or bears any causal relationship to collateral development, and temporal changes during follow-up could not be evaluated. Third, several potentially relevant variables, including circulating angiogenic cytokines, oxidative stress markers, endothelial progenitor cell function, insulin resistance, and family history of coronary artery disease, were not systematically available and therefore could not be incorporated into the analysis. Ethnicity was also not systematically recorded, precluding assessment of potential differences across ethnic groups. Similarly, a history of recent trauma was not systematically documented and was not assessed as a separate exclusion criterion; therefore, its potential influence on inflammatory markers, particularly hs-CRP, cannot be completely excluded. Specific data on menopausal status and estrogen levels were also unavailable, limiting our ability to assess the potential contribution of estrogen depletion to vascular vulnerability and coronary collateral development. Fourth, collateral circulation was evaluated angiographically using the Rentrop classification, which reflects anatomical collateral filling rather than functional collateral blood flow or tissue perfusion. Fifth, no external validation cohort was available, and the reported cut-off value should therefore be regarded as exploratory. Finally, excluding patients who had received lipid-lowering therapy within the preceding three months improved biological homogeneity but may limit the generalizability of the findings to the broader CTO population. Detailed CTO-related anatomical and clinical parameters, including the J-CTO score, prior myocardial infarction, and exact pressure gradients, were not systematically recorded, leaving potential residual confounding related to CTO characteristics.

## 5. Conclusions

In this retrospective, single-centre cohort of patients with chronic total occlusion, higher RCII values were independently associated with poorer coronary collateral circulation, and RCII demonstrated greater discriminatory performance than remnant cholesterol, hs-CRP, and SII considered separately. These findings indicate an association rather than causality and should be considered hypothesis-generating. RCII may represent a candidate marker of the combined residual lipid and inflammatory burden associated with coronary collateral development; however, prospective studies and external validation are needed before any clinical application can be considered.

## Figures and Tables

**Figure 1:**
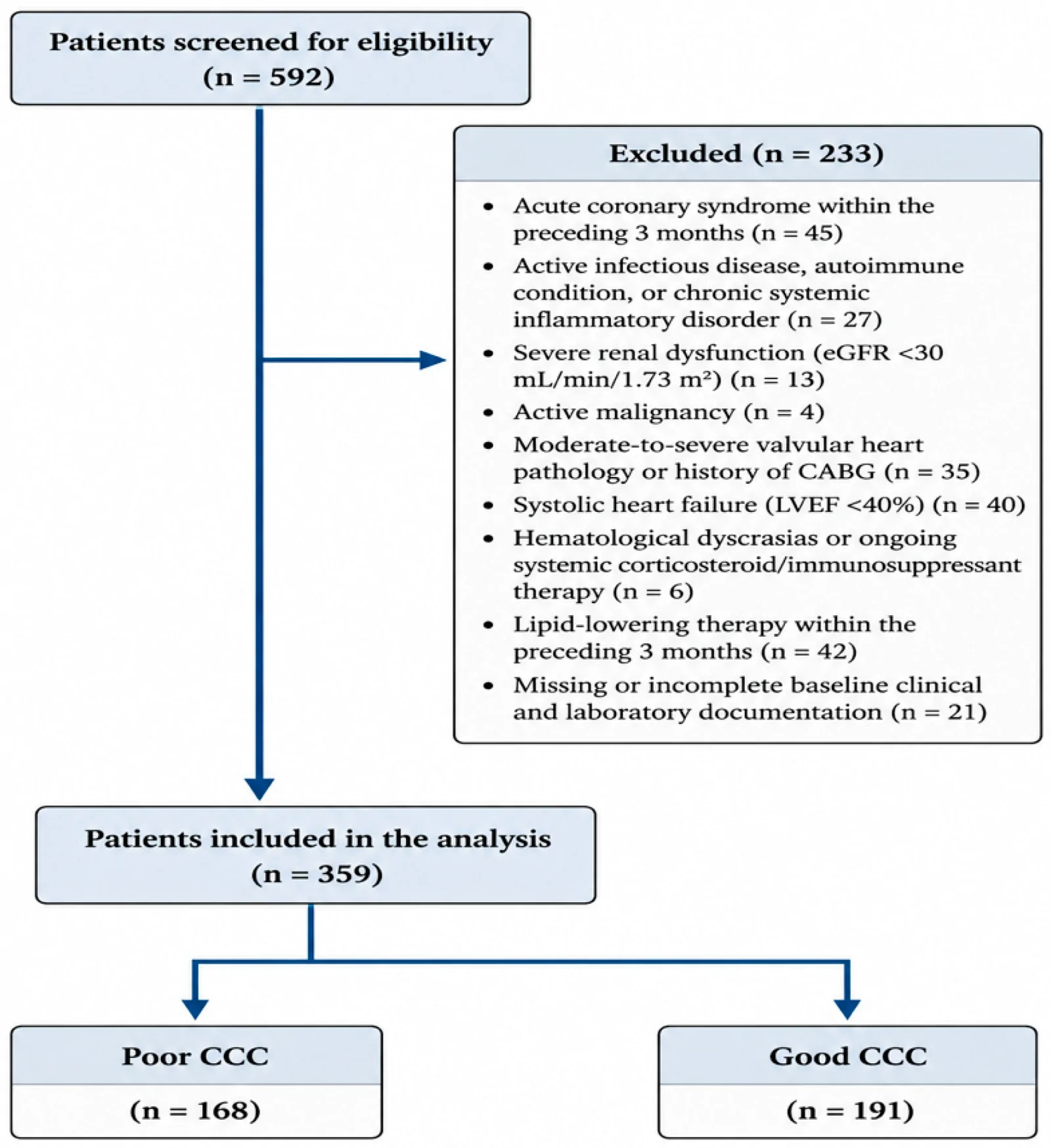
Flow diagram depicting the selection of study participants and their allocation into study groups.

**Figure 2:**
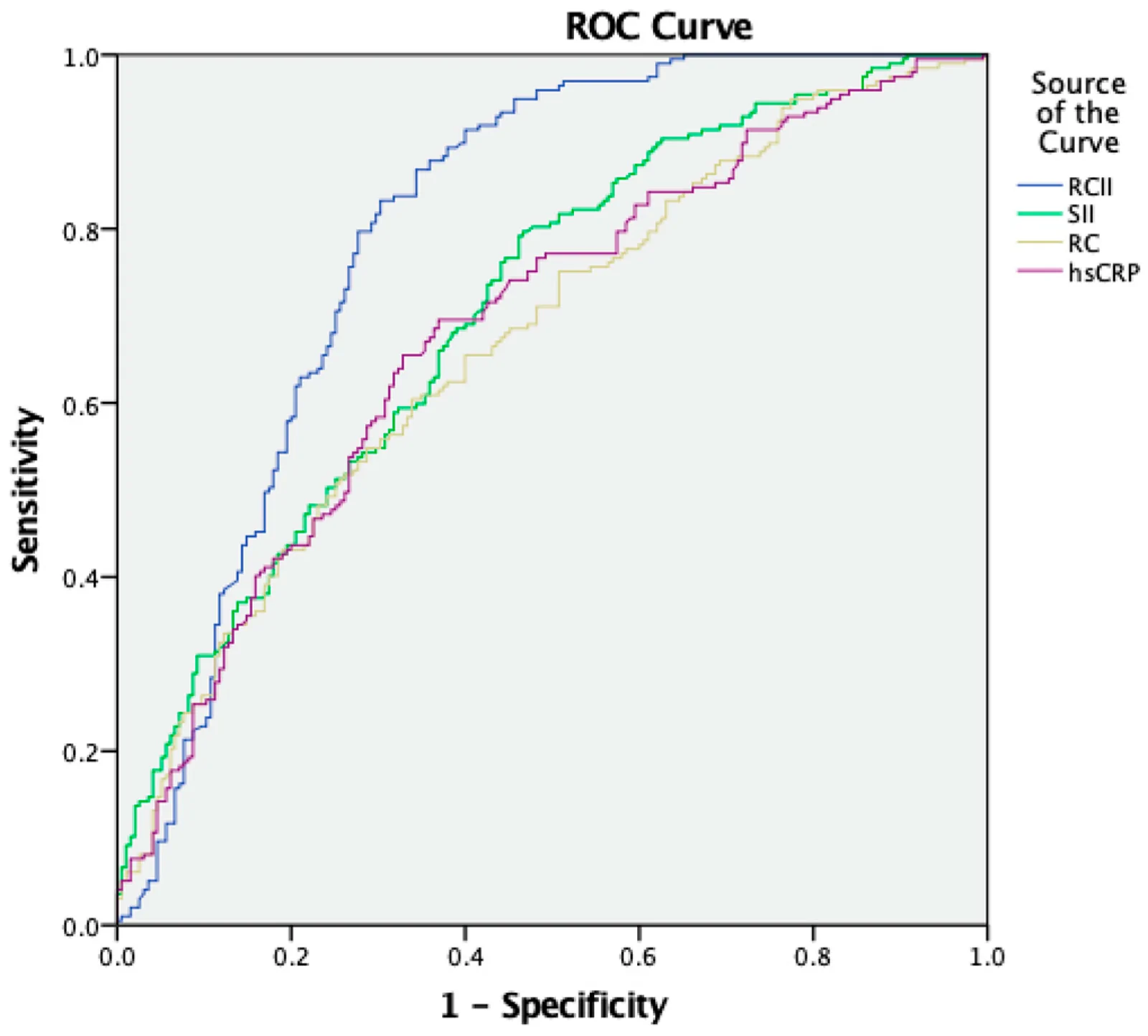
Receiver operating characteristic (ROC) curves of RCII, SII, hs-CRP, and RC for discriminating poor coronary collateral circulation in patients with chronic total occlusion. hs-CRP, high-sensitivity C-reactive protein; RC, remnant cholesterol; RCII, remnant cholesterol inflammation index; SII, systemic immune-inflammation index.

**Table 1:** Baseline clinical, demographic and angiographic characteristics of the groups.

	All (*n* = 359)	Poor CCC (*n* = 168)	Good CCC (*n* = 191)	*p*
Age (years)	66.9 ± 8.4	67.6 ± 8.8	66.3 ± 9.1	0.170
Female sex, n (%)	125 (34.8)	58 (34.5)	67 (35)	0.923
Diabetes mellitus, n (%)	111 (30.9)	59 (35.1)	52 (27.2)	0.106
Ejection Fraction, (%)	52 ± 7.1	51 ± 6.0	52 ± 8.2	0.185
BMI (kg/m^2^)	26.8 ± 2.1	27.1 ± 1.9	26.7 ± 2.2	0.065
Hypertension, n (%)	201 (55.9)	100 (59.5)	101 (52.8)	0.206
Systolic BP (mmHg)	131 ± 19.5	132 ± 20.2	130 ± 18.4	0.323
Diastolic BP (mmHg)	77 ± 10.8	78 ± 9.4	77 ± 10.4	0.339
Smoking, n (%)	144 (40)	70 (41)	74 (38.7)	0.570
ACE-inhibitor/ARB use, n (%)	107 (29.8)	53 (31.5)	54 (28.3)	0.563
Beta-blocker use, n (%)	65 (18.1)	35 (20.8)	30 (15.7)	0.208
**CTO location, n (%)**				
LAD	93 (25.9)	50 (29.8)	43 (22.5)	0.118
Cx	58 (16.2)	33 (19.6)	25 (13.1)	0.092
RCA	208 (57.9)	85 (50.6)	123 (64.4)	0.008
**Number of diseased vessels, n (%)**				
One-vessel disease	50 (13.9)	22 (13)	28 (14.6)	0.760
Two-vessel disease	118 (32.8)	54 (32.1)	64 (33.5)	0.784
Three-vessel disease	191(53.2)	87 (51.7)	104 (54.4)	0.614

BMI, body mass index; Cx, circumflex artery; LAD, left anterior descending artery; RCA, right coronary artery.

**Table 2:** Laboratory and inflammatory parameters of the groups.

	Poor CCC (*n* = 168)	Good CCC (*n* = 191)	*p*
Glucose (mg/dL) (median) [IQR]	121 [71–181]	110 [58–169]	**0.042**
Creatinine, mg/dL	1.01 ± 0.21	0.99 ± 0.12	0.272
HbA1c	6.9 ± 1.3	6.3 ± 1.0	**<0.001**
Total cholesterol (mg/dL)	206 ± 31	185 ± 33	**<0.001**
Low density lipoprotein cholesterol (mg/dL)	121 ± 26	112 ± 31	**0.003**
High density lipoprotein cholesterol (mg/dL)	41 ± 13	49 ± 10	**<0.001**
Triglycerides (mg/dL)	156 ± 44	135 ± 52	**<0.001**
Hemoglobin, g/dL	13.5 ± 1.2	13.3 ± 1.7	0.201
Neutrophil count, 10^3^/μL	6.4 ± 1.4	5.6 ± 1.7	**<0.001**
Platelets count, 10^3^/μL	241 ± 70	227 ± 48	**0.027**
Lymphocyte count, 10^3^/μL	2.0 ± 0.4	2.1 ± 0.7	0.096
NLR	3.9 ± 0.5	2.9 ± 1.0	**<0.001**
Remnant cholesterol (mg/dL)	44 ± 9	24 ± 11	**<0.001**
RCII	19.7 ± 4.7	6.5 ± 2.4	**<0.001**
hs-CRP (mg/L)	4.2 ± 0.9	2.1 ± 0.8	**<0.001**
SII (median) [IQR] (×10^3^)	1235 [795–2165]	757 [363–1609]	**<0.001**

hs-CRP, high-sensitivity C-reactive protein; NLR, neutrophil/lymphocyte ratio; RCII, remnant cholesterol inflammation index; SII, systemic immune-inflammation index. Bold *p*-values indicate statistically significant results (*p* < 0.05).

**Table 3:** Univariable and multivariable analyses of factors associated with poor CCC.

Variables	Univariate Analysis OR (95% CI)	*p*-Value	Multivariable Model 1 OR (95% CI)	*p*-Value	Multivariable Model 2 OR (95% CI)	*p*-Value
Age	1.018 (0.990–1.047)	0.212	1.030 (0.990–1.070)	0.150	1.028 (0.988–1.069)	0.170
Hypertension	1.310 (0.863–1.988)	0.205	1.240 (0.849–1.789)	0.260	1.280 (0.870–1.880)	0.220
Diabetes mellitus	1.447 (0.925–2.264)	0.106	1.008 (0.730–1.365)	0.960	1.112 (0.833–1.496)	0.450
RCA CTO	0.624 (0.408–0.955)	**0.030**	0.562 (0.335–0.937)	**0.026**	0.695 (0.490–0.990)	**0.043**
Remnant cholesterol	1.425 (1.123–1.808)	**0.004**	1.228 (1.004–1.506)	**0.046**		
hs-CRP	2.715 (1.947–3.785)	**<0.001**	2.018 (1.350–3.007)	**<0.001**		
SII	1.027 (1.014–1.040)	**<0.001**	1.004 (1.001–1.007)	**0.009**	1.003 (1.001–1.005)	**0.003**
RCII	1.487 (1.181–1.872)	**<0.001**			1.120 (1.052–1.199)	**<0.001**

CI, confidence interval; hs-CRP, high-sensitivity C-reactive protein; OR, odds ratio; RCII, remnant cholesterol inflammation index; SII, systemic immune-inflammation index. Bold *p*-values indicate statistically significant results (*p* < 0.05).

## Data Availability

The data presented in this study are available on request from the corresponding author due to privacy restrictions.

## Abbreviations

The following abbreviations are used in this manuscript:

AUC	area under the curve
CAD	coronary artery disease
CCC	coronary collateral circulation
CTO	chronic total occlusion
hs-CRP	high-sensitivity C-reactive protein
NLR	neutrophil-to-lymphocyte ratio
NPAR	neutrophil percentage-to-albumin ratio
PIV	pan-immune-inflammation value
RC	remnant cholesterol
RCII	remnant cholesterol inflammation index
RLP-C	remnant-like particle cholesterol
SII	systemic immune-inflammation index
TIMI	Thrombolysis in Myocardial Infarction

## References

[1] (2024). GBD 2021 Causes of Death Collaborators. Global burden of 288 causes of death and life expectancy decomposition in 204 countries and territories and 811 subnational locations, 1990–2021: A systematic analysis for the Global Burden of Disease Study 2021. Lancet.

[2] Fefer P., Knudtson M.L., Cheema A.N., Galbraith P.D., Osherov A.B., Yalonetsky S., Gannot S., Samuel M., Weisbrod M., Bierstone D. (2012). Current perspectives on coronary chronic total occlusions: The Canadian Multicenter Chronic Total Occlusions Registry. J. Am. Coll. Cardiol..

[3] Seiler C., Stoller M., Pitt B., Meier P. (2013). The human coronary collateral circulation: Development and clinical importance. Eur. Heart J..

[4] Meier P., Hemingway H., Lansky A.J., Knapp G., Pitt B., Seiler C. (2012). The impact of the coronary collateral circulation on mortality: A meta-analysis. Eur. Heart J..

[5] Meier P., Schirmer S.H., Lansky A.J., Timmis A., Pitt B., Seiler C. (2013). The collateral circulation of the heart. BMC Med..

[6] Neshat N., Shayestehyekta H., Moradi M., Frishman W.H., Aronow W.S. (2025). Coronary Collaterals: A Comprehensive Review. Cardiol. Rev..

[7] le Noble F., Kupatt C. (2022). Interdependence of Angiogenesis and Arteriogenesis in Development and Disease. Int. J. Mol. Sci..

[8] Yalcin M.U., Gurses K.M., Aydoğan C., Butun S., Tunçez A., Tezcan H., Ozen Y., Demir K., Aygul N., Kirmizigul M. (2026). Endothelial Activation and Stress Index Predicts Poor Coronary Collateral Development in Chronic Total Occlusion. J. Cardiovasc. Dev. Dis..

[9] Aktaş İ., Bolayır H.A., Karasu M. (2025). Neutrophil Percentage-to-Albumin Ratio as a Predictor of Collateral Circulation in Chronic Total Occlusion. Anatol. J. Cardiol..

[10] Kelesoglu S., Yilmaz Y., Elcık D., Kalay N. (2022). Systemic immune inflammation index: A novel predictor for coronary collateral circulation. Perfusion.

[11] Nacar A.B., Erayman A., Kurt M., Buyukkaya E., Karakaş M.F., Akcay A.B., Buyukkaya S., Sen N. (2015). The relationship between coronary collateral circulation and neutrophil/lymphocyte ratio in patients with coronary chronic total occlusion. Med. Princ. Pract..

[12] Toprak K., Karataş M. (2025). Exploring the impact of pan-immune-inflammation-value and other traditional inflammation-based markers on coronary collateral circulation in patients with chronic total occlusion. BMC Cardiovasc. Disord..

[13] Varbo A., Benn M., Tybjærg-Hansen A., Jørgensen A.B., Frikke-Schmidt R., Nordestgaard B.G. (2013). Remnant cholesterol as a causal risk factor for ischemic heart disease. J. Am. Coll. Cardiol..

[14] Varbo A., Benn M., Tybjærg-Hansen A., Nordestgaard B.G. (2013). Elevated remnant cholesterol causes both low-grade inflammation and ischemic heart disease, whereas elevated low-density lipoprotein cholesterol causes ischemic heart disease without inflammation. Circulation.

[15] Wang K., Wang R., Yang J., Liu X., Shen H., Sun Y., Zhou Y., Fang Z., Ge H. (2022). Remnant cholesterol and atherosclerotic cardiovascular disease: Metabolism, mechanism, evidence, and treatment. Front. Cardiovasc. Med..

[16] Gao A., Liu J., Liu Y., Hu C., Zhu Y., Zhou Y., Han H., Zhao Y. (2022). Increased estimated remnant-like particle cholesterol is associated with impaired coronary collateralization in patients with coronary chronic total occlusions. Diabetol. Metab. Syndr..

[17] Doi T., Langsted A., Nordestgaard B.G. (2023). Dual elevated remnant cholesterol and C-reactive protein in myocardial infarction, atherosclerotic cardiovascular disease, and mortality. Atherosclerosis.

[18] Ma Q.-L., Du L.-L., Peng J. (2025). Association of Remnant Cholesterol Inflammation Index with Cardiovascular Risks and All-Cause Mortality in Individuals with Diabetes or Prediabetes. Diabetes Metab. J..

[19] Chen J., Wu Q., Liu H., Hu W., Zhu J., Ji Z., Yin J. (2025). Predictive value of remnant cholesterol inflammatory index for stroke risk: Evidence from the China Health and Retirement Longitudinal Study. J. Adv. Res..

[20] Aktaş İ., Yaşar E., Uçkaç K. (2026). Association Between the Remnant Cholesterol Inflammation Index and Cardiac Syndrome X. Diagnostics.

[21] McEvoy J.W., McCarthy C.P., Bruno R.M., Brouwers S., Canavan M.D., Ceconi C., Christodorescu R.M., Daskalopoulou S.S., Ferro C.J., Gerdts E. (2024). 2024 ESC Guidelines for the management of elevated blood pressure and hypertension. Eur. Heart J..

[22] American Diabetes Association Professional Practice Committee (2025). 2. Diagnosis and Classification of Diabetes: Standards of Care in Diabetes—2025. Diabetes Care.

[23] Ferrer R., Mansilla A., Tondo M., Blanco-Vaca F. (2023). Remnant cholesterol: New outcomes highlight its potential as a clinically useful cardiovascular risk factor. J. Lab. Precis. Med..

[24] Wang Y., Bi L., Li Q., Wang Q., Lv T., Zhang P. (2025). Remnant cholesterol inflammatory index and its association with all-cause and cause-specific mortality in middle-aged and elderly populations: Evidence from US and Chinese national population surveys. Lipids Health Dis..

[25] Faber J.E. (2025). Genetic determinants of insufficiency of the collateral circulation. J. Cereb. Blood Flow Metab..

[26] Red-Horse K., Das S. (2021). New Research Is Shining Light on How Collateral Arteries Form in the Heart: A Future Therapeutic Direction?. Curr. Cardiol. Rep..

[27] Zhang B., Li Y., Peng A., Liu C., Lin J., Feng Y., Wan J. (2024). Association between the pan-immune-inflammation value and coronary collateral circulation in chronic total coronary occlusive patients. BMC Cardiovasc. Disord..

[28] Lähteenvuo J.E., Lähteenvuo M.T., Kivelä A., Rosenlew C., Falkevall A., Klar J., Heikura T., Rissanen T.T., Vähäkangas E., Korpisalo P. (2009). Vascular endothelial growth factor-B induces myocardium-specific angiogenesis and arteriogenesis via vascular endothelial growth factor receptor-1- and neuropilin receptor-1-dependent mechanisms. Circulation.

[29] Hein T.W., Singh U., Vasquez-Vivar J., Devaraj S., Kuo L., Jialal I. (2009). Human C-reactive protein induces endothelial dysfunction and uncoupling of eNOS in vivo. Atherosclerosis.

[30] Eyiol A., Ertekin B., Acar T. (2024). The association between systemic inflammatory indices and prognosis in acute coronary syndrome. Ann. Clin. Anal. Med..

[31] Eyiol A., Eyiol H., Yilmaz A., Sari H. (2026). Pan-Immune-Inflammatory Value (PIV) and HALP Score as Independent Prognostic Indicators in Acute Coronary Syndrome Patients. J. Clin. Med..

[32] Liu L., Wen T., Zheng X.Y., Yang D.G., Zhao S.P., Xu D.Y., Lü G.H. (2009). Remnant-like particles accelerate endothelial progenitor cells senescence and induce cellular dysfunction via an oxidative mechanism. Atherosclerosis.

[33] Nakamura T., Uematsu M., Yoshizaki T., Kobayashi T., Watanabe Y., Kugiyama K. (2020). Improvement of endothelial dysfunction is mediated through reduction of remnant lipoprotein after statin therapy in patients with coronary artery disease. J. Cardiol..

